# Assessing the Evidence for Maternal Pertussis Immunization: A Report From the Bill & Melinda Gates Foundation Symposium on Pertussis Infant Disease Burden in Low- and Lower-Middle-Income Countries

**DOI:** 10.1093/cid/ciw530

**Published:** 2016-11-02

**Authors:** Ajoke Sobanjo-ter Meulen, Philippe Duclos, Peter McIntyre, Kristen D. C. Lewis, Pierre Van Damme, Katherine L. O'Brien, Keith P. Klugman

**Affiliations:** 1 Bill & Melinda Gates Foundation, Seattle, Washington; 2 World Health Organization, Geneva, Switzerland; 3 National Centre for Immunisation Research and Surveillance of Vaccine Preventable Diseases, University of Sydney, Westmead, New South Wales, Australia; 4 Centre for Evaluation of Vaccination, Vaccine and Infectious Diseases Institute, University of Antwerp, Belgium; 5 Department of International Health, International Vaccine Access Center, Johns Hopkins Bloomberg School of Public Health, Baltimore, Maryland

**Keywords:** pertussis, infants, neonates, maternal immunization, vaccines

## Abstract

Implementation of effective interventions has halved maternal and child mortality over the past 2 decades, but less progress has been made in reducing neonatal mortality. Almost 45% of under-5 global mortality now occurs in infants <1 month of age, with approximately 86% of neonatal deaths occurring in low- and lower-middle-income countries (LMICs). As an estimated 23% of neonatal deaths globally are due to infectious causes, maternal immunization (MI) is one intervention that may reduce mortality in the first few months of life, when direct protection often relies on passively transmitted maternal antibodies. Despite all countries including pertussis-containing vaccines in their routine childhood immunization schedules, supported through the Expanded Programme on Immunization, pertussis continues to circulate globally. Although based on limited robust epidemiologic data, current estimates derived from modeling implicate pertussis in 1% of under-5 mortality, with infants too young to be vaccinated at highest risk of death. Pertussis MI programs have proven effective in reducing infant pertussis mortality in high-income countries using tetanus-diphtheria-acellular pertussis (Tdap) vaccines in their maternal and infant programs; however, these vaccines are cost-prohibitive for routine use in LMICs. The reach of antenatal care programs to deliver maternal pertussis vaccines, particularly with respect to infants at greatest risk of pertussis, needs to be further evaluated. Recognizing that decisions on the potential impact of pertussis MI in LMICs need, as a first step, robust contemporary mortality data for early infant pertussis, a symposium of global key experts was held. The symposium reviewed current evidence and identified knowledge gaps with respect to the infant pertussis disease burden in LMICs, and discussed proposed strategies to assess the potential impact of pertussis MI.

Successful implementation of many initiatives targeted to achieving the Millennium Development Goals has led to an average annual decrease of approximately 5% in under-5 mortality over the last 2 decades. However, the rate of decrease in neonatal mortality is lagging at approximately 3% annually, and neonatal mortality now accounts for about 45% of all under-5 mortality [1, 2]. The latest World Health Organization (WHO) estimates are of 2.68 million neonatal deaths in 2015, 86% (2.31 million) of which were in low- and lower-middle-income countries (LMICs) [2]. In addition, there were an estimated 2.6 million previously untracked stillbirths in 2015, >98% of which occurred in LMICs [3]. Following up on the unfinished agenda of the Millennium Development Goals, the newly created Sustainable Development Goals aim to end all preventable under-5 mortality by 2030 [4]. As approximately 23% of neonatal deaths and potentially 10%–50% of stillbirths are thought to be due to infectious causes [1, 5, 6], maternal immunization (MI) is a promising intervention to potentially protect infants from vaccine-preventable mortality in their first months of life. The Bill & Melinda Gates Foundation launched an initiative to evaluate the potential impact of MI in LMICs, with specific focus on 5 eligible infectious diseases. Vaccines are already available for 3 of these (influenza, pertussis, and tetanus), and for the other 2—respiratory syncytial virus (RSV) and group B *Streptococcus*—vaccines specifically designed for MI are currently in clinical development. MI may also bring direct benefits to the mother during and after pregnancy [7] and could be integrated as a complement to other initiatives in the continuum of mother–infant care (Figure 1). Maternal immunization has been most effectively implemented within the maternal neonatal tetanus elimination program. This program has immunized >150 million women of childbearing age globally, as 1 of 4 program components that have together achieved >90% reduction of neonatal tetanus worldwide [8].

**Figure 1.**
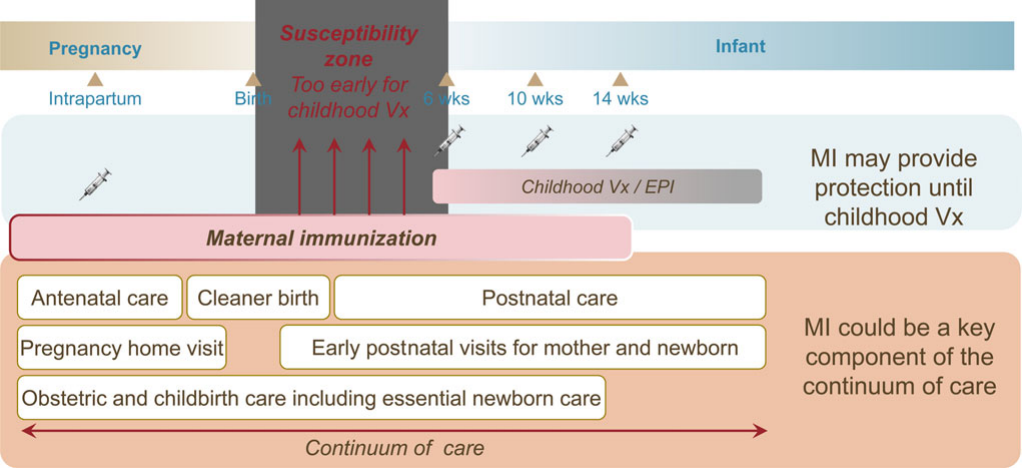
The continuum of care during pregnancy and through the neonatal and early childhood periods. Abbreviations: EPI, Expanded Programme on Immunization; MI, maternal immunization; Vx, vaccination.

One pathogen causing severe disease in early infancy, which can be targeted by MI, is *Bordetella pertussis*. Case fatality rates for pertussis disease are highest in the period between birth and 6–8 weeks of age. If the pathogen is circulating in the community, protection during this period relies on passive transmission of maternal antibody, as this is too early for infants to derive any protection from even the first dose of pertussis vaccine received through the Expanded Programme on Immunization (EPI) vaccination series. MI against pertussis is already recommended as an additional strategy in some high-income countries (HICs) using acellular pertussis (aP) vaccines in their infant series, where a clear, although small, residual burden of infant mortality was documented [9, 10]. Some Pan American Health Organization (PAHO) countries still using whole-cell pertussis (wP) vaccines for infants have also started to recommend MI with aP-containing vaccines [11]. Data on the infant pertussis disease burden in LMICs is less clear, and assessing the need and justification for pertussis MI in such countries requires adequate knowledge of the vaccine-preventable infant pertussis mortality burden. Furthermore, only aP vaccines and not wP vaccines are suitable for vaccinating pregnant women, and reduced-dose diphtheria-tetanus-acellular pertussis (Tdap) vaccines currently used in pertussis MI programs in HICs are probably cost-prohibitive for routine use in LMICs. Programmatic and vaccine delivery considerations for pertussis MI will need to be further evaluated. Funding the development of lower-cost vaccines, and assessing the feasibility and strategic role of routine MI programs, particularly in LMICs, will require a strong evidence-based investment case to ensure that decisions about limited resources are targeted to achieve significant reductions in infant mortality.

This article reports on a symposium of key technical leaders in pertussis infant disease and epidemiology, vaccinology, and policy that was convened in Atlanta, Georgia, in February 2016. The meeting reviewed data from published and ongoing studies on infant pertussis epidemiology in LMICs, reported in detail in the accompanying articles in this supplement, with ascertainment of severe early infant pertussis in LMICs a key focus. The symposium objectives were to identify remaining gaps in the evidence base for pertussis disease in infants <6 months of age in LMICs and to recommend potential strategies to address these gaps to inform MI vaccine research and development and eventually inform pertussis MI policy recommendations for LMICs.

## EFFECTIVENESS OF MATERNAL IMMUNIZATION AGAINST PERTUSSIS

**Figure 2.**
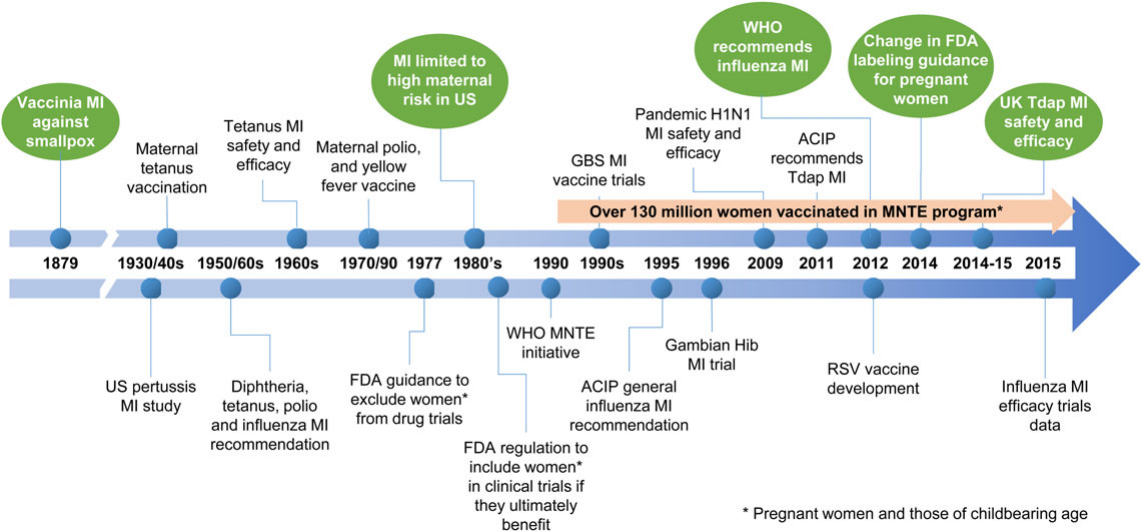
The history of maternal immunization [12]. Abbreviations: ACIP, Advisory Committee on Immunization Practices; FDA, US Food and Drug Administration; GBS, group B *Streptococcus*; Hib, *Haemophilus influenzae* type b; MI, maternal immunization; MNTE, maternal neonatal tetanus elimination; RSV, respiratory syncytial virus; Tdap, reduced-dose tetanus-diphtheria-acellular pertussis vaccine; WHO, World Health Organization.

Maternal immunization has a long history (Figure 2); pertussis MI was recommended by the US Advisory Committee on Immunization Practices (ACIP) in 2011 [9], as a result of an increase in pertussis-related mortality in very young infants in the United States [13]. The previously recommended “cocooning” strategy of vaccinating postpartum mothers and other family members of newborn infants had proven too difficult to implement effectively, as it requires complete coverage of contacts [9]. Safety surveillance in the United States has shown pertussis MI vaccination to be safe [14, 15], but vaccine uptake has been low and variable, and is currently estimated at <50% [14, 16].

Following an increase in pertussis-related hospitalizations and deaths in infants <3 months of age, the United Kingdom implemented an emergency pertussis MI program in 2012 using the Tdap–inactivated polio vaccine that was already in use in their preschool program and so available in sufficient quantity [17]; in 2014 a recommendation was made to continue with the strategy until 2019, at which time a further assessment would be undertaken. Safety surveillance of >20 000 pregnancies in the United Kingdom has shown no increased risk of a range of potential pregnancy-related adverse events [18]. Pregnant women, when advised by their healthcare provider that vaccination in pregnancy is recommended and provided free of charge, have readily accepted the vaccine [19]. The UK MI program has resulted in a significant reduction of neonatal pertussis, with an estimated vaccine effectiveness of about 90% [20, 21]. Other countries including Argentina [11], Australia [22], Canada [23], and New Zealand [24] have recently adopted this strategy.

One potential concern with pertussis MI is that maternal antibodies may blunt the newborn infant's response to routine pertussis vaccinations. There is evidence of blunting in studies from the United States, the United Kingdom, Belgium, and Vietnam [25–28], but the effect is not apparent following toddler booster doses [20], and the clinical significance is unknown. These observations are mainly based on mothers and infants who received aP vaccines; the effect needs to be investigated in LMICs following aP vaccination of pregnant women who have previously received wP vaccination, and in their infants when they receive wP-based EPI vaccines [29].

## CURRENT EVIDENCE FOR PERTUSSIS DISEASE BURDEN

As detailed in this supplement, the true nature of the global pertussis disease burden in infants remains uncertain as methodological limitations, different case definitions, difficulties in diagnosis, and low rates of laboratory confirmation may all lead to underdiagnosis and underreporting. Despite differences in study designs, there is consistent evidence across studies for circulation of pertussis in all geographies, but limited evidence for severe disease and mortality, especially in infants. Effective immunization against pertussis since the 1940s has drastically decreased the global disease burden, most noticeably in HICs with high coverage of diphtheria-tetanus-whole cell pertussis vaccine (DTwP) [30]. In many of these countries, wP vaccines were replaced with less reactogenic, but more expensive, aP vaccines in the early 1990s. In view of new evidence of the advantages of wP, the WHO recommends that LMICs should continue to use wP for primary immunization [31].

### Global Pertussis Disease Burden

**Figure 3.**
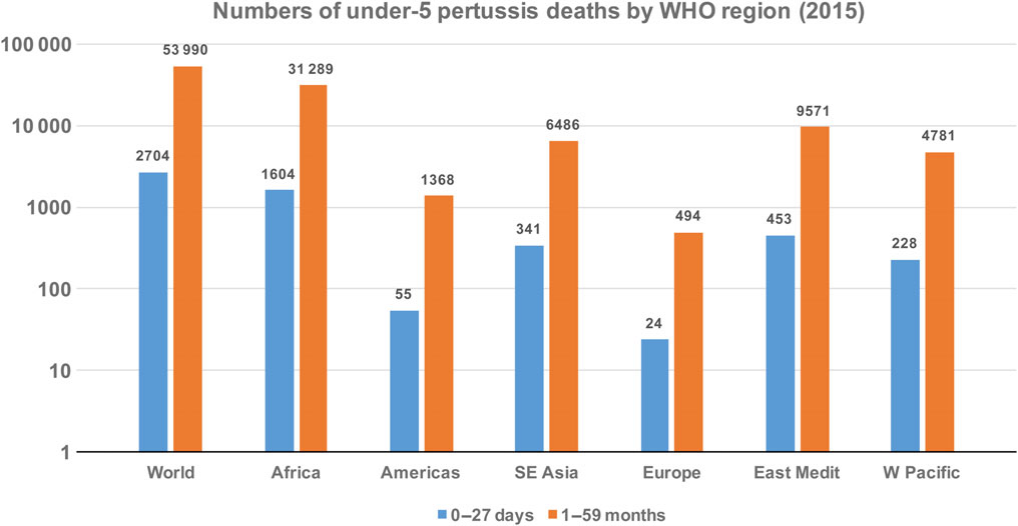
Under-5 pertussis deaths in the different World Health Organization (WHO) regions in 2015, during and after the first month of life [33].

Although pertussis remains endemic worldwide, it is not widely recognized as a public health problem. In the absence of adequate laboratory diagnostic capacity, almost universal outside high-income settings, cases are almost exclusively identified on clinical grounds alone. At the global level in 2015, according to WHO modeling-based estimates but not based on actual incidence data, there were approximately 56 700 pertussis deaths in children <5 years (2700 among neonates and 54 000 among 1- to 59-month-olds), accounting for approximately 1% of all under-5 deaths [32]. Of these deaths, more than half occur in Africa (Figure 3) [34].

When relying on clinical syndrome for diagnosis, some fatal cases of pertussis are unlikely to be recognized as such, especially among very young infants who may not present with cough. Even with cough, the diagnosis may be confused with viral infection or bacterial pneumonia [35]. This may also lead to clinicians failing to obtain laboratory confirmation by culture or polymerase chain reaction (PCR). Early studies of UK data, where pertussis morality was reported at a 6-fold lower rate than the United States, suggested that only approximately 10% of infant pertussis mortality was reported as such based on routine clinical diagnosis, otherwise being recorded as generic conditions such as respiratory disease or pneumonia [36, 37].

### Pertussis Disease Burden in HICs

Despite underdiagnosis and underreporting, the most reliable pertussis surveillance data are from HICs, where laboratory-confirmed surveillance systems and standardized case definitions are applied. Such surveillance has shown a substantial decrease in pertussis incidence following implementation of routine diphtheria-tetanus-pertussis (DTP) immunization in the 1940s, but a transient resurgence of disease occurred in some countries such as the United Kingdom in the late 1970s and early 1980s, due to underimmunization associated with the parental reluctance to use the wP vaccines before aP vaccines became available [31]. A more gradual and persistent increase in pertussis disease has been observed in some aP-using countries since the 1980s, even in countries with high infant vaccine coverage, and several have observed a marked resurgence since 2011–2012 [38–40]. Many factors have been proposed to be responsible for this increase, including low vaccine coverage, lack of booster immunizations, improvements in surveillance and diagnostic methods, and genetic changes in the circulating organisms [40]. Another likely factor is the waning of the protective immunity afforded by the aP vaccines, which is of shorter duration than that elicited by wP vaccines [40]. Waning immunity following childhood aP vaccination as well as protection against disease but not against infection allows infection of adolescents and adults in whom diagnosed cases may only represent the tip of the iceberg of true pertussis incidence. Such continuing circulation of the bacterium may manifest as severe disease and mortality largely in the vulnerable neonate and infant populations that are too young to be immunized and so suffer the major part of the pertussis mortality burden. Several studies have demonstrated that close family members, often the mother, are the source of infection in infant pertussis cases [41–43]. The role of siblings is more variable [44], but a recent US study found a shift to siblings as the main reservoir of infection [45].

### Pertussis Disease Burden in LMICs

The overall high pertussis burden in Africa, and Asia, especially compared with Europe and the Americas (Figure 3), is probably due to deficiencies in vaccination coverage [46]. However, there is a paucity of surveillance data from the region, as well as evidence of underreporting in the surveillance that does exist [32]. Much of the available information was obtained to assess the impact of the introduction of the EPI vaccines, which confirmed high pertussis incidence and case fatality rates pre-EPI, particularly in young infants [47, 48]. The major impact on disease incidence following introduction of the EPI, especially in infants <6 months of age [48], decreased the interest in maintaining surveillance. Evidence of continued circulation of pertussis and infections in highly vaccinated African populations exists, but the absolute magnitude and relationship to DTP coverage remain unclear [49–52]. Studies routinely using PCR to identify *Bordetella pertussis* in confirmation of respiratory specimens from children with cough illness showed that there are still cases occurring, but whether this is discordant with EPI coverage is unknown [51, 52].

In South Asia the reported incidence of pertussis following EPI introduction is low, but outbreaks have been reported among isolated, unimmunized populations in India [53], a country known to contain multiple subpopulations with much lower EPI coverage than the country mean. While serosurveillance studies demonstrate that the organism is still circulating and is responsible for cough illness in adults in wP-using Thailand and South Korea [54–56] and aP-using Malaysia and Taiwan [55], there is no evidence of severe disease in infants.

Latin America has also experienced a recent resurgence in pertussis, which has manifested mainly in young infants with no or incomplete vaccinations [57]. This has been associated with increased incidence among adolescents and adults who may be the source of the infant infections. Following a large number of pertussis-related deaths in 2011, about 60% of which occurred in infants <2 months of age, Argentina introduced pertussis MI in 2012 [11]. Resurgence of pertussis in Parana, Brazil, stimulated a retrospective observational and cross-sectional study of 3451 confirmed pertussis cases from 2007 to 2013, which identified that most cases, and 95% of deaths, were in infants <3 months of age [58]. The majority occurred in unvaccinated or incompletely vaccinated infants. Studies in other Latin American countries (Chile, Colombia, and Mexico) also reported that most pertussis-related deaths occurred in the first year of life [57]. A Peruvian study used PCR analysis to detect *B. pertussis* in samples from almost 600 children <5 years of age hospitalized for undiagnosed acute respiratory illness from January 2009 to September 2010, before the pertussis epidemic in 2012–2013 [59]. High prevalences of *B. pertussis* (19%) and RSV (17%) PCR positivity were detected; most *B. pertussis*–positive cases were <1 year of age, and many were <3 months of age. Analysis of case records did not reveal any clinical symptoms specifically associated with pertussis, illustrating how pertussis in young infants and children may go undiagnosed in a period not associated with a pertussis epidemic.

This accumulated evidence across different geographies and study methodologies indicates circulating disease in LMICs without a significant burden of severe disease or mortality in infants. It is unclear whether this indicates a continued period of low incidence following the introduction of EPI, or whether cases are not being diagnosed or reported. Underreporting may be due to inadequate clinical case definitions, lack of laboratory confirmation, or misidentification by other diseases. Generally, there are higher levels of coverage with a first dose of DTP but lower coverage and less timeliness with the second and third DTP doses [60].

## RECENT EPIDEMIOLOGIC SURVEILLANCE

### Prospective Population-, Community-, and Hospital-Based Pertussis Surveillance Studies

The symposium reviewed 3 recent studies in Pakistan, Zambia, and South Africa, which are described in detail in this supplement [61–63]. Zambia and Pakistan use wP vaccines for routine EPI, whereas South Africa has introduced aP vaccines in 2009 [64]. The South African study was a hospital-based respiratory and sepsis surveillance study, and results could be projected to the population level as it included most hospitalizations in the local area. The Zambian and Pakistan studies were both population-based community surveillance studies and used a broad and sensitive a priori syndromic case definition to ascertain cases. All 3 studies used a more specific Centers for Disease Control and Prevention (CDC) case definition (Table 1) for the determination of either probable cases and confirmed cases to analyze the study data after the study was completed [65]. All 3 studies detected pertussis disease in young infants, peaking at 1–2 months of age, but with no hospitalizations or deaths observed in the 2 community surveillance studies.

**Table 1.** Case Definitions (2014) Used in Post Hoc Analysis of Data From Pakistan, Zambian, and South African Studies

Infant Meets the US CDC Case Definition of *Either*:
Probable case Acute cough illness of any duration, with at least 1 of the following symptoms: ○ Paroxysms of coughing ○ Inspiratory whoop ○ Posttussive vomiting ○ Apnea (with or without cyanosis) *AND* Meets 1 of the following criteria: ○ PCR-positive test for pertussis ○ Contact with a laboratory-confirmed case of pertussis
Confirmed case ­Meets the clinical case definition of cough illness of ≥2 weeks with at least 1 of the following symptoms, accompanied by a positive PCR test for pertussis or contact with a laboratory-confirmed case of pertussis: ○ Paroxysms of coughing ○ Inspiratory whoop ○ osttussive vomiting ○ Apnea (with or without cyanosis) *OR* Acute cough of any duration with isolation of *Bordetella pertussis* from a clinical specimen

Source: Centers for Disease Control and Prevention [64].

Abbreviations: CDC, Centers for Disease Control and Prevention; PCR, polymerase chain reaction.

### Studies on Infant Sepsis and Pneumonia

Although not designed to study infant pertussis specifically, 2 complementary, large, multicountry surveillance studies on infectious causes of respiratory illness and sepsis have been performed that provide some data on pertussis incidence in LMICs.

### The Aetiology of Neonatal Infections in South Asia Study

The Aetiology of Neonatal Infections in South Asia (ANISA) study [66] is a population-based surveillance study in mothers and their infants up to 59 days of age. Detection of *Bordetella* species was one of 28 pathogens in reverse-transcription PCR analyses of blood and combined nasopharyngeal/oropharyngeal swab samples. A total of >60 000 neonates were enrolled at birth from a rural site in Bangladesh, 2 sites in Pakistan, and 2 sites in India, and monitored at scheduled visits for possible serious infection using a case definition based on the WHO Integrated Management of Childhood Illness definitions. Controls were selected using an algorithm to match age and time of enrollment of cases. Although there were some variations in overall rates across sites with available data (the Odisha, India, sites results were still pending), *Bordetella* positivity was found at similarly low rates at all sites of Bangladesh, Pakistan, and India (Sylhet, 1.6%; Matiari, 0.5%; Vellore, 2.7%) except for the Karachi site of Pakistan (13.5%). Controls were selected from the same birth cohort as the cases from among the registered births in the surveillance according to an algorithm that roughly matched their sample collection age and date to the age/enrollment date of the cases. Notably, positivity rates among the controls were similar to the cases, across all sites. There were approximately 3000 neonatal deaths among the cohort, 70% in the first week of life, with samples available from about 10% of these cases. PCR testing did not reveal any differences in *Bordetella* prevalence between fatal and nonfatal cases.

### The Pneumonia Etiology Research for Child Health Study

The Pneumonia Etiology Research for Child Health (PERCH) study was a case-control study of hospitalized pneumonia in children aged 1–59 months from 2011 to 2014, in 7 countries (Gambia, Kenya, Mali, South Africa, Zambia, Bangladesh, and Thailand); infants <28 days of age were not included in PERCH [67]. The results reported in the companion article [68] show that only a small fraction of cases and deaths were *B. pertussis* positive, occurring mainly in infants aged 1–5 months. However, among these young pertussis-infected pneumonia cases, the case fatality ratio was substantially higher than for RSV, a common pathogen identified in pneumonia cases. Most pertussis cases in PERCH occurred in children too young to have started or completed their pertussis vaccine schedule. Furthermore, pertussis positivity was not exclusively identified in cases; a small number of control subjects were also found to be infected with pertussis.

Although neither of these 2 complementary studies was designed specifically to interrogate the pertussis disease burden, case definitions used were broadly inclusive to identify pertussis, and they found consistently low rates of *B. pertussis* in the included sites in Africa and Asia.

### Studies Conducting Secondary Specimen Testing for Pertussis

In 3 controlled clinical trials conducted in Mali, Nepal, and South Africa [69], pregnant women were randomized to receive inactivated influenza vaccination or a comparator, and their infants were followed up through 6 months of life. Nasopharyngeal specimens were collected in infants who exhibited symptoms of a respiratory illness or influenza-like illness and, in South Africa, those who presented or were hospitalized with any respiratory illness. These specimens were tested post hoc for *B. pertussis*, and the standardized CDC pertussis case definition (Table 1) was applied to determine pertussis cases. While the results of these studies demonstrated *B. pertussis* infections, with the majority of cases occurring among infants <4 months of age and a comparatively higher number of cases occurring in the South African infants, there were few cases of severe pertussis disease and no hospitalizations among infants in Mali and Nepal.

### Surveillance Studies in West Africa

An ongoing population-based surveillance of pneumococcal disease is monitoring for the bacterial etiology in children admitted for suspected pneumococcal disease in children from 2 to 59 months of age following the introduction of pneumococcal conjugate vaccines in The Gambia [70]. As this has recently been expanded to include all admitted children including neonates, there is an opportunity to evaluate the contribution of pertussis by applying appropriate case definitions and testing specimens. A second ongoing study of approximately 5000 neonatal admissions to the main Gambian teaching hospital during 2009–2013 is designed to assess the causes of neonatal mortality [71]. Published data do not indicate that there is a significant infant pertussis disease burden in The Gambia or Senegal following introduction of the EPI [47, 49]. However, because a substantial fraction (34%) of admissions are for likely severe bacterial infections, of which 95% are undiagnosed, further investigation may reveal a previously undiagnosed pertussis disease burden.

## PROSPECTIVE PERTUSSIS STUDIES

### Child Health and Mortality Prevention Surveillance

Because most neonatal and early infant deaths in LMICs do not occur in hospital environments, and there is limited laboratory capacity to perform diagnostic tests, it is difficult to ascertain the true causes of infant deaths. Child Health and Mortality Prevention Surveillance (CHAMPS), a major Bill & Melinda Gates Foundation– and CDC-supported initiative to establish a health surveillance network aimed at preventing childhood mortality in sub-Saharan Africa and Southeast Asia, is intended to fill this gap. Currently under development, the intent of CHAMPS is to establish the means to perform surveillance and facilitate locally run studies that will encourage use of data to shape policy. The core of the project is to perform minimally invasive tissue sampling postmortem, with the intent of obtaining samples within 24 hours of death. Causality-assignment algorithms and expert panel review will then be applied systematically to identify a definitive cause of death. Initial results are expected from a subset of sites within 2 years. This project has the potential to provide novel insights into the causes of death, but the challenges to conduct the project are substantial.

### Vaccine Manufacturers

Vaccine manufacturers, notably the producers of Tdap vaccines, are also seeking to better understand the role that maternal pertussis vaccines play in pertussis epidemiology in wP-using countries. The manufacturers are looking to understand the resurgence in pertussis incidence and potential impact of MI. They recognize the knowledge gaps in both in HICs and in LMICs on the response to pertussis vaccine in the pregnant woman, the transmission of immunity to the infant, and the possible blunting of infant responses to routine pertussis vaccination. The current use of their products in the United Kingdom and United States will be sufficient to generate the necessary data for countries that use aP vaccine in infants. However, their involvement to support studies to generate data from LMICs using wP-based EPI vaccines remains unclear. Such studies could be useful to provide the required data to inform the use of their products in MI programs in countries with both aP and wP infant vaccine programs.

### Summary of Known Disease Burden in wP-Using LMICs

There may be underascertainment of pertussis infections, but the limited evidence currently available does not support any significant underestimation of infant pertussis deaths. However, to have a clear understanding of the character of infant pertussis outcomes, investment is required to increase laboratory capacity and local expertise to perform case ascertainment with standardized methodologies to strengthen the evidence of the actual pertussis infant mortality burden in LMICs. Clinical diagnosis requires standardized case definitions that are optimized for detecting pertussis in infants, that recognize the different presentations of the disease in young children and neonates, and that are sensitive enough to capture the full spectrum of pertussis disease. Further information, including community-based infant mortality surveillance studies in LMICs, will be required to clarify the characteristics and risk factors for pertussis infant mortality in such settings.

## THE ROLE OF INFANT PERTUSSIS DISEASE BURDEN IN INFORMING MATERNAL IMMUNIZATION POLICY RECOMMENDATIONS

Data generated from epidemiologic studies will only be useful if used to inform policies and their implementation through programs designed to meet the identified unmet medical need, particularly for MI. Such programs are technically supported by guidance and technical support from the WHO and its regional offices.

### World Health Organization

Although large-scale immunization programs have resulted in a steep decline in pertussis incidence, the disease remains endemic globally, including in LMICs and even those countries with high infant DTP vaccine coverage. The Strategic Advisory Group of Experts (SAGE) on Immunization pertussis working group has investigated drivers for disease resurgence in 19 HICs and LMICs using aP and wP vaccines and concluded that infant vaccination with either type of pertussis vaccine provides disease protection: 1 dose provides about 50% protection against severe disease, with >80% protection after 2 doses [31]. However, immunity wanes with aP and is probably responsible for the resurgence observed in Australia (with increased PCR-based diagnostic testing as a contributing factor), Portugal, the United States, and the United Kingdom. The current WHO priority to decrease the global pertussis disease burden is to support improvements in the DTP coverage through EPI: All children should be vaccinated, with timely vaccination from 6 weeks of age and with subsequent doses 4–8 weeks apart to ensure 90% coverage with 3 doses by 6 months of age. Countries currently using wP vaccines should continue to do so, while those using aP should consider the need for additional boosters [31].

The WHO recommends that countries with high pertussis morbidity and mortality in those too young to be vaccinated consider implementing pertussis MI, as this is the most efficient additional strategy beyond routine infant immunization coverage, with 1 dose of Tdap administered in the second or third trimester and at least 15 days before delivery [31]. More epidemiologic data are required to inform policy decisions on the potential value of pertussis MI in LMICs. Specifically, there is an urgent need to improve pertussis disease burden surveillance in LMICs, with assessments of the impact of infant immunization. Particular focus must be on fatalities in infants <1 year of age, especially in neonates. These data must be combined with realistic assessments of the potential impact of MI on both neonatal and overall pertussis burdens, and of the current ability to implement MI programs with projections of the likely uptake. Issues of potential blunting of the infant immune response to wP and the effectiveness of current pertussis vaccines also need to be addressed. The necessity of vaccinating for each pregnancy also needs to be assessed, as well as the impact on affordability and cost-effectiveness [31].

### Pan American Health Organization

PAHO has recently transitioned from focusing on childhood immunization to whole-life immunization, to include adolescent, adult, and senior citizen immunizations, with MI at the core of this new approach. The 45 countries in the PAHO region variously use wP and aP vaccines in their infant schedules, and most report vaccine coverage >90% with 3 doses of DTP, but with major regional variations. Typically, there are 15 000–30 000 cases of pertussis reported in the region per year, but during the epidemic in 2012 there were almost 72 000 cases reported. Cases are reported in all age groups, but 21%–25% are in children <1 year of age.

PAHO has established a working group with the WHO, CDC, and pertussis experts to regionalize the implementation of SAGE MI recommendations, which are currently available for tetanus-low dose diphtheria and influenza in addition to the pertussis recommendation noted above. In the absence of robust disease burden data, PAHO currently recommends pertussis MI to be limited to outbreak settings (as for hepatitis A, hepatitis B, yellow fever, and meningococcal vaccines). The decision to introduce pertussis MI at the country level is made by the country on the recommendation of its National Immunization Technical Advisory Group (NITAG). As of the date of the symposium, 14 of the 45 PAHO member countries have introduced routine Tdap for MI since 2012, with regional coverage ranging from 36% to 100% (Maria Cristina Pedreira, Regional Advisor, PAHO, personal communication, June 2016). Surveying these countries revealed that the criteria considered in the decision-making process included data on disease burden, disease severity, cost-effectiveness, vaccine safety and efficacy, and national data, as well as WHO/PAHO recommendations, but also at times there was a strong influence of the US ACIP recommendations.

PAHO intends to strengthen surveillance systems in the region to evaluate the pertussis disease burden and vaccination impact in children and pregnant women to inform decisions to introduce MI. Improvements are still necessary to ensure timely and high routine infant immunization coverage with 3 doses of DTP. Justifying the extension of pertussis MI policy to routine immunization of pregnant women would require the generation of more evidence on the impact among infants in the region, not only on disease incidence but also on the possible blunting of immune responses to recommended infant vaccinations (pertussis and other vaccines), vaccine effectiveness, and vaccine safety, noting the mixed use of aP and wP vaccines.

## CONCLUSIONS AND RECOMMENDATIONS (Table 3)

In the EPI era, pertussis remains an endemic disease globally, albeit at much lower rates compared with the prevaccine era. There is evidence of a resurgence in some countries using aP vaccines (United States, United Kingdom), despite high levels of infant pertussis vaccination coverage [39, 40]. The implementation of MI with TdaP vaccines in these countries has proven highly effective, resulting in substantial decreases in neonatal pertussis mortality [20, 21, 25]. In LMICs, which are largely using wP in their infant programs, current data from multiple studies in several countries using different study designs consistently show persistent circulation of pertussis and identify cases of pertussis infection, but have not found cases in young infants requiring hospitalization or causing death.

Gaining the necessary local, national, and international support to implement pertussis MI in LMICs requires more robust data that characterize the burden of severe early infant pertussis. Information will also be required on the potential impact of pertussis MI on reducing infant mortality, together with estimates of the likely incremental benefit of pertussis MI vs alternatives in these settings, such as the WHO-driven strategy of improving timely and complete EPI vaccination coverage. Case detection must be improved to collect accurate information on the pertussis burden and thereby improve model estimates of disease burden. This may require the use of different case definitions and improvements in the quality and availability of pertussis diagnostic tests, accompanied by training of local laboratory personnel as well as training of clinicians to recognize pertussis in young infants. Point-of-care diagnostics need to be improved to enable confirmation of pertussis in clinically suspected cases, and diagnostic laboratory capacity needs to be enhanced, to strengthen the required evidence base.

Specific studies of the role of pertussis in infant deaths are needed as essential inputs into disease burden modeling to assist policy decision making, together with the information indicated in Table 2 [72] to assess the risk (or number) of vaccine-preventable pertussis-attributable deaths in a population and so allow cost-effectiveness calculations of pertussis MI. Studies performed over a longer duration will be useful to contextualize the burden of pertussis-associated deaths against a country's natural pertussis disease cycle, to reduce uncertainties of whether incidence is measured at a peak or trough of the cycle. Ongoing studies focused on preventable infant mortality can potentially be leveraged to identify causal criteria for pertussis-related mortality and death attribution, but community-based mortality surveillance studies will have to be performed, determining the frequency with which infant home deaths are associated with pertussis and not counted in facility-based pertussis burden studies. Studies investigating the infant pertussis disease burden in LMICs should have a known population denominator and need to use standardized case definitions specific for pertussis in infants that are suitable for application in LMICs.

**Table 2.** Data Required for Modeling the Cost-effectiveness of Pertussis Maternal Immunization

Data Required for Cost-effectiveness Modeling and Analyses		
Domain	Subdomain	Comment
Disease burden	Risk of pertussis-attributable death	Influenced by case definitions, diagnostic methods, and method of attribution. Nonfatal morbidity also important in middle-income countries.
Economic burden	Disease treatment costs	
Prevention effectiveness	Proportion of pertussis-attributable deaths preventable by MI	Accounts for the proportion of deliveries and pertussis cases among premature infants and the vaccine coverage at various gestational ages
	Age distribution of pertussis-attributable deaths	
	Infant vaccine coverage	Disease burden that can be averted by MI may be impacted by proportions of infants receiving no wP, on-time wP, or delayed wP vaccination.
	MI vaccine efficacy	Needs to take into consideration preterm delivery and other factors that may impact transfer of passive immunity, in addition to duration of protection.
	MI vaccine coverage	Requires estimation of proportions of women who can realistically be expected to receive timely MI.
Program costs	System, program and delivery costs associated with MI, including the programmatic capacity constraints and risk mitigation.	

Source: [72].

Abbreviations: MI, maternal immunization; wP, whole-cell pertussis.

**Table 3.** Main Takeaways From the Symposium on Pertussis

Main Takeaways
In the vaccine era, there is a more substantial reduction in the incidence of severe pertussis than in the pre-EPI era, but pertussis remains endemic. Resurgence observed in aP-using countries mainly affects very young infants. The degree of resurgence does not contribute significantly to the overall burden of childhood mortality. Pertussis maternal immunization is highly effective against this infant burden in high-income countries.
The burden of vaccine-preventable infant pertussis in LMICs still requires further investigation if a case is to be made to support introduction of pertussis MI: Available data indicate a consistent low level of pertussis incidence, with few hospitalization or deaths. This may indicate that the impact of EPI introduction is continuing to maintain low levels of incidence in all ages or that there is significant underascertainment of severe infant pertussis. Modeling indicates that effective vaccination of older age groups protects infants.
An investment case for pertussis MI in LMICs must consider disease and vaccine: Pertussis-related mortality burden must be more accurately quantified, with identification of vaccine-preventable cases by age, and assessment of whether cases were vaccinated or not. A known denominator is required to quantify the magnitude of disease. Assumptions of probable and achievable coverage with MI must be realistic, and considered in relation to other factors such as premature birth. Cost-effectiveness assessments must include costs of pertussis MI, including vaccine, program, and delivery costs, and must be balanced against costs of preventing other diseases with high mortality burden.
Robust epidemiologic data are required from LMICs: Consistent case definitions of pertussis applicable to the local situation need to be applied. Standardized case definitions, including apnea in infants, are required for sensitivity of diagnosis of pertussis and of severe disease. Studies need to be performed in LMICs with investment in the local infrastructure to enable local laboratory confirmation of pertussis diagnoses. Background information on true rates of coverage and punctuality of EPI must be available. Local and regional healthcare providers must be involved so generated data are used to drive local policies and recommendations.
Priority may be given to improving current EPI programs to ensure higher and punctual coverage with EPI DTwP vaccine: Potential impact of immune blunting needs to be understood in countries using DTwP for routine infant immunization.

Abbreviations: aP, acellular pertussis; EPI, Expanded Programme on Immunization; DTwP, diphtheria-tetanus-whole cell pertussis vaccine; LMICs, low- and lower-middle-income countries; MI, maternal immunization.

While current evidence does not indicate a substantial infant pertussis mortality burden in LMICs, the strategies to address the existing burden cannot be further assessed without additional data. Other eligible platforms for clarifying the infant pertussis disease burden in LMICs so that the most effective additional strategies can be prioritized include demographic surveillance systems, serological studies, and studies with the potential to conduct postmortem specimen testing for pertussis. Serological evaluation of population sources of infections and related modeling are potentially important tools in understanding the risk for infant pertussis disease and mortality. This includes the potential identification of a correlate of protection against infant pertussis death using population-based seroepidemiological data in pregnant women in settings with adequate active pertussis surveillance in their infants, as well as in animal models.

If a more complete disease burden evidence base indicates a role for pertussis MI in LMICs, implementation and uptake of MI will only be feasible if safe, effective, and affordable aP vaccines become available, as the current vaccines are very probably cost-prohibitive for LMICs. MI delivery approaches and programmatic challenges will also have to be addressed. Early analyses suggest that a dose cost under $1.00 may be necessary for the vaccine to pose good value [72]. The availability of such vaccines, including potential combination vaccines that protect against additional MI disease targets, will help policy makers to better assess the cost-effectiveness of MI as a complementary intervention to control infant pertussis mortality along with current strategies that focus on maximizing infant and childhood vaccination against pertussis.
